# A Novel Anaerobic Gravity-Driven Dynamic Membrane Bioreactor (AnGDMBR): Performance and Fouling Characterization

**DOI:** 10.3390/membranes12070683

**Published:** 2022-06-30

**Authors:** Yingfei Pu, Zihan Fu, Tingting Li, Yucheng Chen, Zhongbo Zhou

**Affiliations:** 1 College of Resources and Environment, Southwest University, Chongqing 400715, China; puyingfei0104@163.com (Y.P.); fzh18943761099@163.com (Z.F.); 17774993674@163.com (T.L.); chenyucheng@swu.edu.cn (Y.C.); 2 Chongqing Engineering Research Center of Rural Cleaner Production, Chongqing 400715, China

**Keywords:** membrane bioreactor, nylon net, non-woven fabric, irreversible fouling, cake layer

## Abstract

Despite numerous studies undertaken to define the development and significance of the dynamic membrane (DM) formed on some coarse materials, the optimization of reactor configuration and the control of the membrane fouling of anaerobic dynamic membrane bioreactor (AnDMBR) need to be further investigated. The aim of this study was to design a novel anaerobic gravity-driven dynamic membrane bioreactor (AnGDMBR) for the effective and low-cost treatment of municipal wastewater. An 800 mesh nylon net was determined as the optimal support material based on its less irreversible fouling and higher effluent quality by the dead-end filtration experiments. During the continuous operation period of 44 days, the reactor performance, DM filtration behavior and microbial characteristics were studied and compared with the results of recent studies. AnGDMBR had a higher removal rate of chemical oxygen demand (COD) of 85.45 ± 7.06%. Photometric analysis integrating with three-dimensional excitation–emission matrix fluorescence spectra showed that the DM effectively intercepted organics (46.34 ± 16.50%, 75.24 ± 17.35%, and 66.39 ± 17.66% for COD, polysaccharides, and proteins). The addition of suspended carriers effectively removed the DM layer by mechanical scouring, and the growth rate of transmembrane pressure (TMP) and the decreasing rate of flux were reduced from 18.7 to 4.7 Pa/h and 0.07 to 0.01 L/(m^2^·h^2^), respectively. However, a dense and thin morphological structure of the DM layer was still observed in the end of reactor operation and plenty of filamentous microorganisms (i.e., *SJA-15* and *Anaerolineaceae*) and the acidogens (i.e., *Aeromonadaceae*) predominated in the DM layer, which was also embedded in the membrane pore and led to severe irreversible fouling. In summary, the novel AnGDMBR has a superior performance (higher organic removal and lower fouling rates), which provides useful information on the configuration and operation of AnDMBRs for municipal wastewater treatment.

## 1. Introduction

Over the past decades, anaerobic membrane bioreactors (AnMBRs) have been widely investigated for municipal wastewater treatment due to methane production, no aeration, and biomass retention [1,2]. However, some obstacles (i.e., membrane fouling, high cost of the membrane module, and low membrane flux) hinder the extensive practical applications of the AnMBRs [3]. The cake layer fouling was a common phenomenon in AnMBRs, which was caused by the deposition of various foulants such as soluble microbial products and fine particles on the surface of the ultrafiltration/microfiltration (UF/MF) membrane [4,5,6]. The cake layer might be used as the secondary filtration due to physical interception and biodegradation effects [7]. The researchers have attempted to use coarse materials (such as nylon net and non-woven fabric) instead of the UF/MF membrane to support and strengthen the formation of cake layers for enhancing the pollutant removal, which is called the dynamic membrane (DM) [8]. Due to the advantages of the low cost and high flux of DM filtration [7,9,10,11], there is a growing interest in anaerobic dynamic membrane bioreactors (AnDMBRs) for wastewater treatment.

Note that membrane fouling is always a major concern for MBR systems. The accumulation of foulant on the supporting coarse materials is the main reason of membrane fouling for AnDMBRs [7]. Nylon nets [12], stainless steel nets [13], and non-woven fabrics [14] were generally used as support materials due to their lower cost and larger pores [15]. In recent years, many studies have discussed the effect of the different pore sizes of the same materials on the formation of DM and the mitigation of reversible fouling [16,17]. However, limited information on the irreversible fouling accumulated in the pores of supporting materials is available. Irreversible fouling could be removed by chemical cleaning, which will boost the aging of the supporting material and affect the long-term filtration performance of the membrane [18,19]. It is important to understand the formation process of irreversible fouling on the supporting materials. Ersahin, et al. [20] summarized that mono-monofilament filter cloth was more appropriate for a DM filtration system compared to the mono-multifilament woven fabric in AnDMBRs due to less irreversible fouling. Apart from the pore size, the type and structure of the supporting materials greatly affected the performance of DM filtration systems. However, the effect of supporting material properties on the accumulation and development of irreversible fouling remains unclear.

Table S1 shows the performance and membrane fouling with different reactor configurations and supporting materials of AnDMBRs reported in recent years. As far as we know, a continuous stirred tank reactor (CSTR) [13,21] and upflow anaerobic sludge bed (UASB) [8,17] could normally combine with the DM module to set up AnDMBRs [7]. The supporting material was exposed to the supernatant of the sludge bed in the membrane-coupled UASB system [17], which led to denser layers and severer membrane fouling [22]. Different module configurations in CSTRs had different cleaning methods (e.g., backwash [12,23] and taking the membrane out for surface brushing [16,21]), which increased high cost and troublesome operation. The operation mode of the reactor including a pump-driven mode [16] and gravity-driven mode [12] could also affect the fouling behaviors in AnDMBRs [7]. Compared with the latter, the pump-driven mode seemed to be more flexible [7]. However, more stable flux could be maintained over a long time in gravity-driven mode, which contributed to the formation of a more heterogeneous biofilm on the membrane surface [24]. Most importantly, the gravity-driven mode has a lower energy consumption [25]. Previous studies mainly focused on improving pollutants’ removal and filtration performance when designing the reactor configuration and operation mode, without further considerations of the fouling control and irreversible fouling formation of AnDMBRs.

Therefore, the main objective of this study was to design a novel AnGDMBR with simple configuration and in situ mechanical cleaning and then to evaluate the reactor performance and fouling behavior for municipal wastewater treatment. Dead-end filtration tests with different support materials (i.e., nylon net and non-woven fabric) were firstly carried out to determine the optimal material following reactor operation. The retention capacity of the DM layer was evaluated by monitoring the water quality and three-dimensional excitation–emission matrix (EEM) analysis. With the addition of suspended carriers, the reversible and irreversible fouling development in the long-term operation of the AnGDMBR were discussed by analyzing the variations in the transmembrane pressure (TMP) and flux, calculating the membrane fouling rate and the proportion of irreversible fouling as well as scanning electron microscope (SEM) characterization. Microbial communities in the bulk sludge and cake layer of the AnGDMBR were further analyzed by high-throughput sequencing. Overall, the novel reactor was expected to have a superior performance, which could provide useful information on the configuration and operation of AnGDMBRs.

## 2. Materials and Methods

### 2.1. Dead-End Filtration Tests

To find an optimal support material, the filtration behaviors of six different coarse materials (30 g, 50 g, 70 g non-woven fabrics (Jialianda Non-woven Fabric Co., LTD, Dongguan, China) and 600 mesh, 800 mesh, 1000 mesh nylon nets (Xurui Net Industry Co., LTD, Haozhou, China) (details in Table S2) were investigated through the dead-end filtration tests at a constant TMP (30 kPa) (Figure S1). The seeding sludge was taken from the reactor, and the initial total suspended solid (TSS) concentration was kept at 4.0 g/L. The liquid was fully mixed with an electric stirrer at a rate of 300 rpm. The permeate weight was continuously measured and recorded by an electronic balance connecting to a computer. The filtration behaviors of 50 g non-woven fabric and 800 mesh nylon net were further investigated with multiple cycles to check the formation of irreversible fouling. The effluent quality and residual fouling layer of the two materials were analyzed. After each dead-end filtration, the cake layer was removed using a brush with tap water and recorded the images of the membrane surface before and after being cleaned. All the above steps were repeated five times.

The fouling resistance caused by the new net, cake layer, pore-blocking, and organic-bound fouling was evaluated following the formula [26,27] (Equation (1)):

(1) $${R\;}_{t}{=R}_{m}{+R}_{c}{+R}_{b}{+R}_{f}=\frac{TMP}{\;\mu \;\times \;J}$$

where R_t_ is the total resistance (m^−1^); R_m_ is the resistance of new support materials (m^−1^); R_c_ is the cake layer resistance (cleaning with sponges) (m^−1^); R_b_ is the pore-blocking resistance (backwashing with 50 mL pure water at 30 kPa) (m^−1^); R_f_ is the organic-bound fouling resistance (chemical cleaing in 0.3% NaClO solutions) (m^−1^); TMP is the transmembrane pressure as (Pa); *μ* is the water viscosity as (Pa·s); *J* is the membrane flux as (m^3^·m^−2^·s^−1^).

### 2.2. Bioreactor Setup, Operation, and In-Situ Mechanical Cleaning

**Figure 1:**
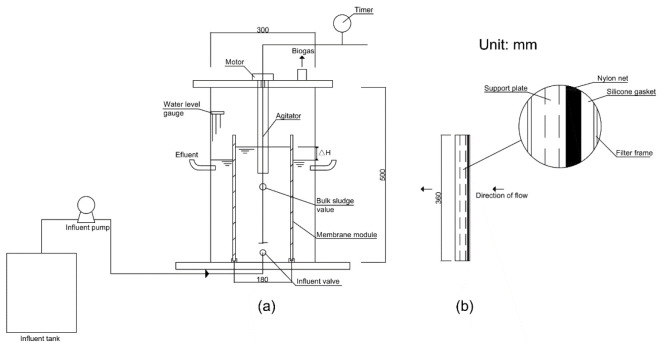
(**a**) Setup of AnGDMBR and (**b**) cross section of membrane modules.

Figure 1 describes the experimental AnGDMBR setup with an effective volume of 30 L, and two membrane modules were inserted vertically into the reactor for solids separation (the related picture of the reactor and module could be found in Figure S2). Several 800 mesh nylon net was served as the support material for checking the formation of DM layers. The effective area of each membrane filter was 0.048–0.064 m^2^. Artificial municipal wastewater with polysaccharide (PS) of 300–350 mg/L, protein (PN) of 40–50 mg/L, and chemical oxygen demand (COD) of 300–500 mg/L was fed to the reactor at a continuous inflow rate of approximately 0.5–0.6 L/h. Table S3 presents details of the wastewater components (all the reagents and chemicals were analytical reagent from the Sinopharm Group, China). The seeding sludge was taken from a lab-scale AnMBR cultured in the laboratory [28]. The reactor was operated at around 33 °C and the pH at ~7.5. The solids retention time (SRT) was 100 d, and the hydraulic retention time (HRT) was 15–18 h.

The filtration process was driven by the head loss (∆H) between the sludge solution and the effluent. With the increase in filtration resistance caused by the biofilm growth on the support material, the water level of the sludge solution would rise to offer a higher transmembrane pressure (TMP). When the ∆H reached 10 cm for the first time, we added the suspended carrier (~20% filling ratio) into the reactor and sped up the mechanical stirring rate (from 100 to 350 rpm) for 30 min to clean the membrane. After physical cleaning and the water level of the sludge solution in the reactor recovered, a new cycle of filtration was continued with the mechanical scouring at 100 rpm. During the operation, the effluent flux and ∆H were regularly monitored. The concentrations of COD, PS, and PN in the influent, supernatant, and effluent were measured to assess the performance of the reactor.

### 2.3. Characterization of the DM Layer

#### 2.3.1. The Retention Capacity of the DM Layer

The supernatant of the bulk sludge was regularly extracted for the measurement of the COD, PS, and PN. The rejection potential of the DM layer was calculated with the concentrations in influent, effluent, and supernatant samples. The analytical methods for these parameters were described in the following Section 2.5.

#### 2.3.2. Fouling Calculation of the DM Layer

The fouling resistance of the DM layer was calculated as shown in Equations (2) and (3):

(2) $$J=\frac{Q}{A_{m}}$$

(3) TMP=∆H × 9.81 × 1000

where *Q* is the permeate flow rate as (m^3^·s^−1^); *A_m_* is the membrane area as (m^2^); ∆H is the head loss as (m).

Membrane flux is taken as the key indicator for membrane fouling [29]. Foulants on the membrane surface that could be removed by physical cleaning are named as reversible fouling (RF), while those that remain in the membrane pore are noted as irreversible fouling (IF). Both of them are total fouling (TF). RF, IF, and TF could be calculated according to the following equations [30]:

(4) $${\mathrm{IF}}_{n}=\frac{J_{p(n-1)}-J_{p(n)}}{J_{p(0)}}$$

(5) $${\mathrm{TF}}_{n}=\frac{J_{p(0)}-J_{f(n)}}{J_{p(0)}}$$

(6) $${\mathrm{RF}}_{n}{=\mathrm{TF}}_{n}-{\mathrm{IF}}_{n}$$

where *J_p_*_(0)_ is the initial membrane flux in the first cycle; *J_p_*_(*n*)_ and *J_f_*_(*n*)_ are the initial flux and final flux of each period.

#### 2.3.3. Fouling Resistance Measurement and Scanning Electron Microscopy (SEM) Characterization of DM Layers

To investigate the fouling resistance distribution and long-term formation behavior of DM layers, the samples were cut from the net after the 44-d operation and further analyzed. One piece was flushed with tap water for cake removal. Meanwhile, one was rinsed with 0.3% NaClO solution for 12 h to remove the pore-blocking and irreversible foulants. After each cleaning step, the fouling resistances, including the cake layer resistance (R_c_), irreversible fouling resistance (R_ir_), unrecoverable fouling (R_un_), and membrane resistance (R_m_) were measured according to the Dancy’s law. Here, the inorganic fouling resistance was not considered, as the synthetic wastewater used was composed of less inorganic matter (Table S3). Afterwards, all of them plus a new net piece were fixed with 2.5% glutaraldehyde for 4–6 h at room temperature, then were washed in 0.05 M phosphate buffer at pH 7.2 and dehydrated in a graded ethanol series. After the vacuum freezer, the samples were coated with gold sputter. Images were made using a Hitachi SU3500 SEM (Tokyo, Japan) at 5 kV.

### 2.4. Microbial Community Analysis

At the end of the operation, the bulk sludge and cake layer were collected to analyze the difference of microbial community between bulk sludge and cake layer. The samples were stored at −80 °C before 16S rRNA gene sequencing. The total genomic DNA was extracted using the OMEGA Soil DNA Kit (D5625-01, Omega Bio-Tek, Norcross, GA, USA) based on the manufacturer’s instructions. A NanoDrop ND-1000 spectrophotometer (Thermo Fisher Scientific, Waltham, MA, USA) was used to examine the DNA concentration and the agarose gel electrophoresis was applied to assess the integrity of DNA. The V4-V5 hypervariable regions of the 16S rRNA genes by PCR were amplified using a unified primer pair of 515F (5′-GTGYCAGCMGCCGCGGTAA-3′) and 926R (5′-CCGYCAATTYMTTTRAGTTT-3′) for bacteria and archaea [31]. PCR products were purified and quantified using Vazyme VAHTSTM DNA Clean Beads (Vazyme, Nanjing, China) and Quant-iT PicoGreen dsDNA Assay Kit (Invitrogen, Carlsbad, CA, USA). On the Illumina MiSeq platform, equimolar-purified amplicons were paired-end sequenced. Raw sequences data were examined with QIIME 2.0 (2019.04) software [32]. After quality filtering, denoising, and merging of sequences, low-quality sequences were eliminated, and high-quality sequences were generated with different amplicon sequences variants (ASVs) by DADA2 with an identification threshold of 70% [33]. Taxonomic characteristics were assigned to ASVs based on the Silva database. QIIME 2.0 was used to calculate alpha diversity (Shannon and Simpson diversity index) and beta diversity (principal coordinates analysis (PCoA)) to estimate microbial community characteristics.

### 2.5. Other Physico-Chemical Analysis

The particle size distribution (PSD) of the bulk sludge was detected by a laser granularity distribution analyzer (S3500, Microtrac Instruments, Montgomeryville, PA, USA). The extracellular polymeric substances’ (EPSs) contents of the bulk sludge samples were regularly measured during the experiment. The heat treatment method was used for the EPS extraction [34]. The concentrations of COD and TSS were measured using the standard method [35]. PS was detected with the phenol-sulfuric acid method at 490 nm with glucose (GR, Sinopharm Group, Shanghai, China) as a standard and PN was analyzed using the Lowry–Folin method with Bovine Serum Albumin (BSA) (GR, Sinopharm Group, China) for the calibration at 750 nm [36,37]. An analyzer (BIOGAS 5000, Geotechnical Instruments Ltd., London, UK) and a wet gas flowmeter were used to monitor the composition and volume of the biogas. The dissolved organic matter (DOM) in influent, effluent, supernatant, and cake layer samples were further characterized by fluorescence spectra analysis (F-7000FL, HITACHI, Japan) [38].

## 3. Results and Discussion

### 3.1. Optimal Support Material for DM Formation and Filtration

**Figure 2:**
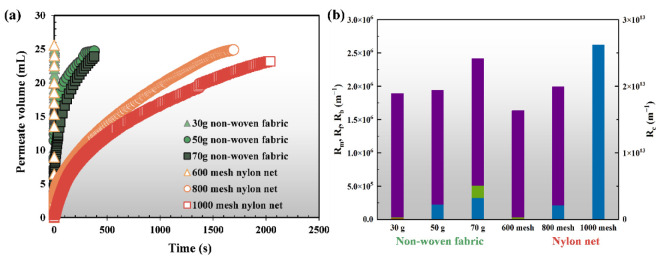
Dead-end filtration curve (**a**) and fouling resistance distribution (**b**) of the different supporting materials. R_c_, R_b_, R_f_, and R_m_ refer to the fouling resistances of cake layers, pore-blocking, organic-bound fouling, and new support filters which were measured through the following cleaning procedures (i.e., cake removal with sponges, backwashing with 50 mL of DI water at 30 kPa, and 0.3% NaClO cleaning for 12 h).

In Figure 2, the filterability of non-woven fabrics was much better than that of nylon nets with the same filtration volume of anaerobic sludge solution due to their shorter filtration time. It was closely related to the pore size of these support materials (Table S2). As reported, a larger pore size of the support material normally has lower fouling resistances [24].

In Figure 2 and Table S4, the cake layer resistances dominated the total fouling resistance (>99%), which could be related to the big particle size of bulk sludge (the volume average diameter of particles was 115.3 μm in Figure S3) and most of the bulk sludge was rejected by the DM layer, leading to higher cake fouling resistances. The 1000 mesh nylon net had the most serious fouling (Table S4) and the largest total fouling resistance due to the smallest pore size (13 μm in Table S2). In Figure S4, the cake layers were easily removed from the support materials after physical cleaning, especially for the nylon net. As reported, a nylon net is the mono-monofilament filter cloth with a smooth surface, which led to the particles tending to be retained by the cake layer on the filter instead of being embedded into the filter pores [20]. In contrast, non-woven fabric is the staple yarn type which has the twisted and hairy structures, resulting in the particles being accumulated into the filter pores and being uneasy to be removed [20]. Note that the R_b_ and R_f_ of the non-woven fabrics accounted for a relatively higher percentage. In particular, the 1000 mesh nylon net with the smallest pore size had the lowest R_f_/R_t_ ratio. Overall, the pore size and structure of support materials played an important role in membrane fouling, and further research should be focused on the irreversible fouling.

**Figure 3:**
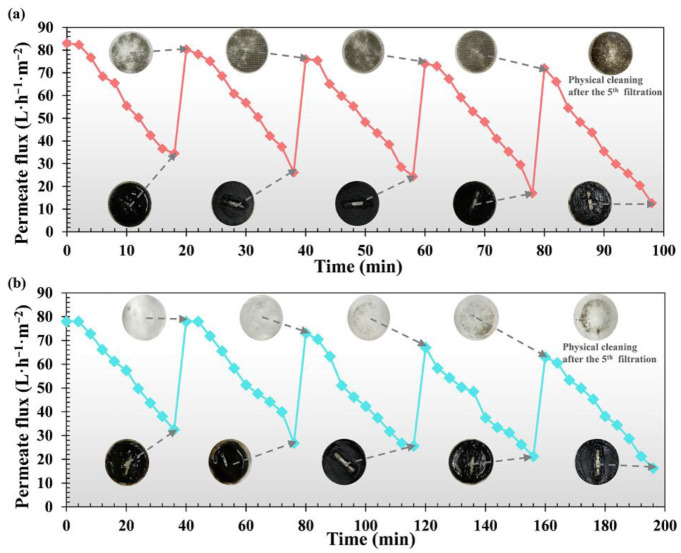
Fouling behavior of (**a**) 50 g non-woven fabric, (**b**) 800 mesh nylon net in multiple filtration-physical cleaning cycle tests. Fouled membrane images were showed after each filtration and physical cleaning.

According to the above results, 50 g non-woven fabric (30 μm) and 800 mesh nylon nets (18 μm) were further used to characterize the irreversible fouling by multiple filtration-physical cleaning cycles. We noted that irreversible foulants gradually accumulated in both of the filter pores (Figure 3). In comparison to the 50 g non-woven fabric, there was less pore-blocking fouling for 800 mesh nylon net after each physical cleaning, indicating that 50 g non-woven fabric could have a higher irreversible fouling resistance. Meng, et al. [39] also determined that the filtration of non-woven material in MBRs for municipal wastewater treatment could be subjected to severe pore fouling. In Figure S5, a gradual increase in the fouling resistance led to permeate flux decline as the irreversible foulants remained and accumulated during the tests. It is worth noting that the fouling resistance of the 50 g non-woven fabric was always higher than that of the 800 mesh nylon net. With an increase in filtration cycles, the retention rates of the particles had an increasing trend due to the formation of the DM layer (Table S5). Mahat, et al. [40] concluded that the DM layer played an important role in removing the organic matter. The effluent quality of the 800 mesh nylon net was obviously higher than that of the 50 g non-woven fabric due to the smaller pore size and the rapid formation of the DM layers [41]. The solids in the permeate showed a decreasing trend, while the solids in the cake layers and solids’ residue amounts in the pore after physical cleaning increased (Table S6). Note that the residual foulants in 50 g non-woven fabric were much heavier than that in the 800 mesh nylon net, indicating that 50 g non-woven fabric had more serious irreversible fouling, which was consistent with the observation in Figure 3. In summary, the performance of the 800 mesh nylon net in terms of membrane fouling and effluent quality during the multiple fouling-physical cleaning cycles was much better than that of 50 g non-woven fabric. We concluded that the 800 mesh nylon net could be much more suitable for the long-term operation of the reactor.

### 3.2. Performance of AnGDMBR

**Figure 4:**
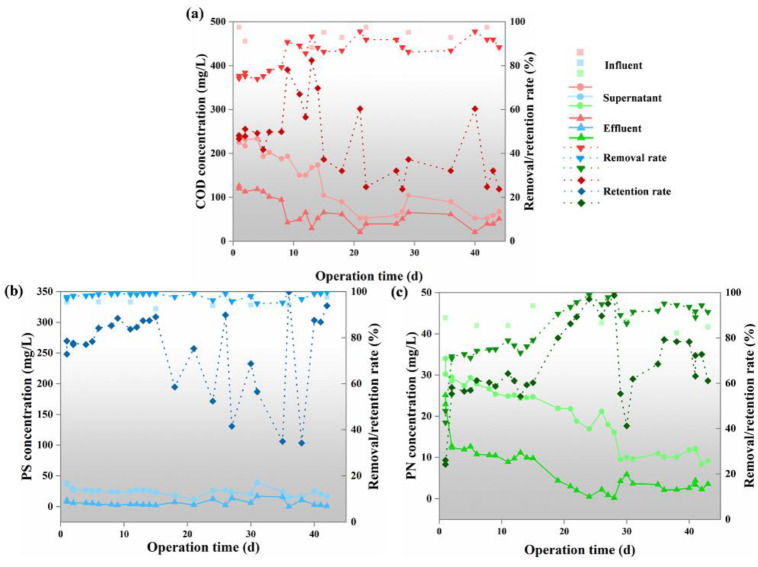
COD (**a**), PS (**b**), and PN (**c**) concentrations in the influent, supernatant, and effluent and the related removal and retention rates of these organics in the AnGDMBR.

After the continuous operation of AnGDMBR for 44 days, the average removal efficiency of COD was 85.45 ± 7.06% (Figure 4a and Table S7). Compared with the other studies in Table S1, the reactor has a better removal rate of COD. The removal efficiencies of PS and PN were 98.15 ± 1.33% and 83.32 ± 13.62%. The biogas production of AnGDMBR was about 4.75 ± 0.40 L/d with a methane yield of 0.24 ± 0.03 L CH_4_/g COD_removed_, which was lower than the theoretical value of 0.35 LCH_4_/g COD_removed_. Yang, et al. [8] found a lower methane yield of 0.05–0.12 L CH_4_/g COD_removed_ under low HRTs (1–8 h) and presented that the lost CH_4_ was dissolved in the effluent at a temperature of 33 °C. We note that the organic matters always had higher concentrations in the supernatant than those in the effluent, suggesting that the organic matters were efficiently retained by the DM layer. The average rejection rates of COD, PS, and PN were 46.34 ± 16.50%, 75.24 ± 17.35%, and 66.39 ± 17.66% (Table S7), which were also much higher compared with the data in the previous reports shown in Table S1. In our study, the pore size of the nylon net used was 18 μm, which is smaller than those in other studies, thus easily developing the DM layer and effectively rejecting the organics [42]. The rejection rates of COD, PS, and PN generally increased with operating time prior to the 15th day (Figure 4). It is plausible to explain that the cake layer and irreversible foulants deposited and accumulated on the membrane as a secondary barrier, enhancing the rejection rates of organics [25]. However, the organic rejection rate declined and became unstable after the 15th day, which could be due to the following two reasons: (i) due to poor transfer of nutrients from the bulk solution, cell lysis, or endogenous decay that occurred at the bottom of DM layers, releasing biopolymers that deteriorated the effluent quality [8,42]; (ii) because of the severe fouling after the 15th day, the frequency of physical cleaning increased to effectively remove the DM layer and recover the flux, which would affect the DM stability and function, resulting in the organics not being intercepted and flowing out in the effluent during/after the physical cleaning.

**Figure 5:**
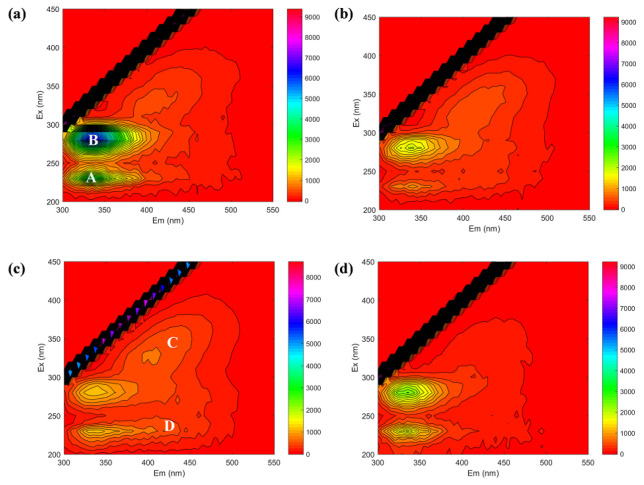
EEM fluorescence spectra of DOM in the influent (**a**), supernatant (**b**), effluent (**c**), and cake layer (**d**). Peaks A, B, C and D mean the aromatic protein-like, tryptophan protein-like, humic acid-like and fulvic acid-like compounds, respectively.

The chemical components (i.e., aromatic protein-like, tryptophan protein-like, humic acid-like, and fulvic acid-like) in the influent, supernatant, effluent, and cake layer in the AnGDMBR were further characterized by the EEM spectra. Figure 5 shows that two protein-like peaks located at the excitation/emission wavelengths (Ex/Em) of 230 nm/335–340 nm (peak A) and the Ex/Em of 280 nm/335–340 nm (peak B) were clearly identified from all spectra [43]. The other two new peaks were presented in the supernatant and effluent rather than the influent and cake layer, which are associated with humic acid-like (Peak C) and fulvic acid-like (Peak D) substances [43]. The fluorescence intensity (FI) of peak A and peak B in the supernatant and effluent were lower than those in the influent, and they had much stronger FI in the cake layer (Table S8), suggesting that protein-like substances could be biodegraded and have a higher molecular weight that is rejected by the cake layer [8]. The FI of peak C and D in the supernatant and effluent were higher than those of the influent and cake layer, probably due to their lower molecular weight and biodegradability [44]. It was also deduced that the humic acid-like and fulvic acid-like substances were probably released from the cell death and endogenous decay in sludge and flowed out in the effluent.

### 3.3. DM Formation and Filtration Behavior

**Figure 6:**
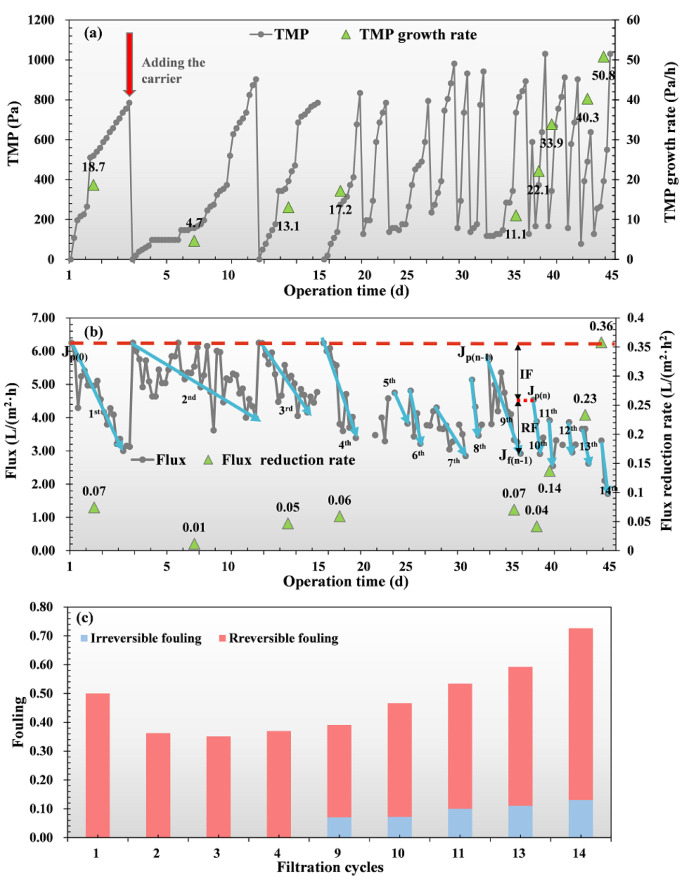
The AnGDMBR in the long-term operation: (**a**) variations in the transmembrane pressure (TMP); (**b**) changes in the flux, and (**c**) effect of filtration cycles on membrane fouling (i.e., reversible, irreversible, and total fouling).

In Figure 6a,b, due to the gravity driven filtration, the TMP increased rapidly from the initial 0 to 800 Pa during the first start-up (the first 3 days), owing to the DM formation and the membrane flux decreased from 6.25 to 3.00 L/(m^2^·h). The moving carrier was added for physical cleaning and mechanical scouring to remove the cake layer and recover the membrane permeability. It is noted that, after the addition of the carrier, the TMP growth rate and the flux reduction rate decreased from 18.7 to 4.7 Pa/h and 0.07 to 0.01 L/(m^2^·h^2^), indicating that adding the carrier is an effective method for causing a reduction in membrane fouling [45]. Compared with other research results in Table S1, it was found that the membrane fouling rate of the reactor was lower in this work, which could be greatly affected by the bioreactor operation (such as influent characteristics, HRT, physical cleaning methods), temperature, and other operating factors [8,13,46,47]. In our study, the nylon net with smaller pore sizes was used and operated under lower TMP and fluxes, leading to faster development of the DM layer with less compact [8]. Plus, the mechanical scouring with the addition of suspended carriers effectively prevented the excessive accumulation of DM layers. Altogether, the high effluent quality and low fouling rate could be achieved in the AnGDMBR system.

However, there was still a general rising trend of TMP and a decreasing trend of the flux in the following operation period. It was reported that the irreversible fouling residuals were retained in the pore of the supporting net after physical cleaning [46]. We note that the irreversible fouling gradually accumulated in the nylon net during the long-term filtration (Figure 6c), resulting in a sharp increase in TMP and a distinct decrease in flux. In Figure 6c, the irreversible fouling continued to rise and was accounted for about 17.90% of the total fouling at the end of the experiment, indicating that irreversible fouling played an essential role in the filtration process of DM systems.

Although it was found that more EPS was produced at a lower temperature, leading to faster membrane fouling [48], Pan, et al. [13] showed a higher resistance at mesophilic condition compared with room temperature, which was due to the fouling induced by proteins and β-ɒ-glucopyranose polysaccharides [49]. However, we note that the fouling resistance in the report of Tang, et al. [23] was lower than that in Pan, et al. [13], with the same temperature in Table S1, indicating that temperature could be not the only factor affecting membrane fouling resistance. In this reactor, the total membrane fouling resistance (3.2 × 10^7^ m^−1^) was minimum compared with others in Table S1, which could be probably due to the smaller compact structure of the DM layers that operated under a lower pressure and flux. In Table 1, the R_t_ was mainly contributed by R_c_ (66.5%) and R_ir_ (24.8%), which led to an increase in the TMP and a decrease in the flux in this study (Figure 6). Li, et al. [47] also found that the irreversible fouling (16.4% of the total fouling resistance) accumulating into the membrane pores in the end of the experiment could be an important reason for increasing the TMP of a nylon net filter module (average pore size of 90 μm) in the MBR. Here, R_m_ (6.2%) and R_un_ (2.5%) were minimal and could be negligible, indicating that the chemical procedures were effective in removing irreversible fouling.

### 3.4. Morphological Structures of DM Layers Characterized by SEM Analysis

**Figure 7:**
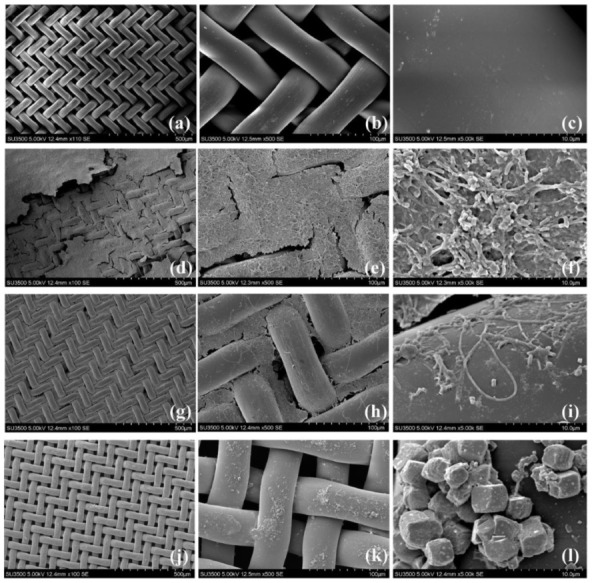
Scanning electron microscope (SEM) images for the biofilms on the support materials of a new nylon net (**a**–**c**) and used membrane after fouling (**d**–**f**), after physical cleaning (**g**–**i**), and after chemical cleaning (**j**–**l**).

As shown in Figure 7a, the virgin nylon net had a smooth surface and clear pores. After the membrane was fouled, a large number of filaments intertwined together and formed a thin and dense biofilm to completely block the pores (Figure 7d), leading to a higher fouling resistance. A closer observation of the fouling membrane (Figure 7f) showed that numerous micrococci were attached to the intertwined filaments, enhancing the adhesion of cell-to-cell and cell-to-surface and forming a dense structure [19]. After removing the surface layer with physical cleaning, it was clear that there were still plenty of filaments tightly adhering into the inner structure of the membrane, although some net pores were opened (Figure 7g). The membrane was soaked into the 0.3% sodium hypochlorite solution, and the remaining biofilms and organic matters in the pores could be easily removed (Figure 7j) [19]. It can be seen in Figure 7l that there were some inorganic particulates deposited on the membrane surface, which might be a result of the treatment with 0.3% sodium hypochlorite solution. Based on the above results and other research summaries [19,50], the formation process of DM could be deduced: at the beginning of the operation, some foulants with filaments such as the skeletons formed inside the net pores and gradually blocked the pores. The surface of the supporting net was covered by the thin biofilms (Figure 7d). Although the cake layer was physically removable, there were still irremovable foulants remaining in the pore of the support nylon net, and they gradually accumulated after repeated physical cleaning (Figure 7g). The biofilms with filaments that formed into the pores of the support nylon net became extremely adhesive and resisted to the mechanical scouring, resulting in a rapid increase in TMP and a significant decrease in flux (Figure 6).

### 3.5. Microbial Community Analysis of Bulk Sludge and Cake Layer

**Figure 8:**
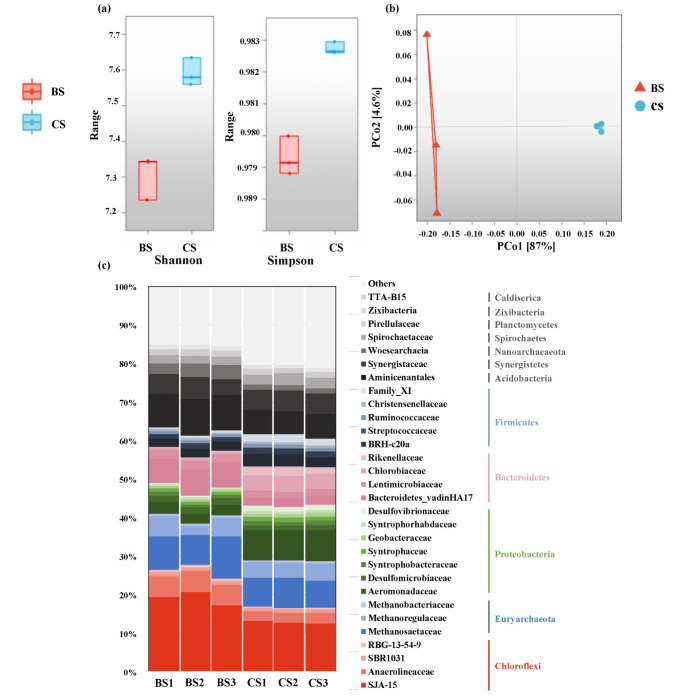
(**a**) Shannon and Simpson indexes of bulk sludge and cake layer samples. (**b**) Principal coordinate analysis (PCoA) plot analysis of community differences between bulk sludge and cake layer samples. (**c**) Relative abundances of the phylum (top 12) and family (top 30) in bulk sludge and cake layer samples.

As shown in Figure 8a, Shannon and Simpson indexes revealed significantly different richness and evenness of the species for bulk sludge and cake layer (*p* = 0.05) [51]. According to the PCoA analysis on community compositions by the Bray–Curtis distances, the bulk sludge samples were obviously separated from those of cake layer samples, displaying that they had different microbial communities (Figure 8b). In Figure 8c, Chloroflexi (16.54–27.70%), Euryarchaeota (10.71–14.74%), Proteobacteria (7.21–14.62%), Bacteroidetes (9.34–10.19%), and Firmicutes (5.33–8.04%) were the dominant phyla in the bulk sludge and cake layer, which was similar to other studies [52,53,54]. A high homology was shown by the main microorganisms in the two community samples of the bulk sludge and cake layer. Based on the previous study [42], it can be inferred that the microorganisms in the cake layer were mainly derived from bulk sludge. Compared with bulk sludge (7.48 ± 0.35%, 9.60 ± 0.30%, and 5.33 ± 0.18%), the cake layer had a higher enrichment of Proteobacteria, Bacteroidetes, and Firmicutes, accounting for 14.26 ± 0.26%, 10.16 ± 0.23%, and 8.04% ± 0.51%, but a low proportion of Chloroflexi and Euryarchaeota (16.58 ± 0.16% and 12.20 ± 0.11%), which, in the bulk sludge, were 26.01 ± 1.51% and 14.02 ± 0.25%. Ma, et al. [53] and Cayetano, et al. [55] also presented similar results in AnDMBRs and indicated that these communities might be enriched on the net in response to the oligotrophic environment in the cake layer. Chen, et al. [56] reported that Chloroflexi could degrade metabolites and dead cells, indicating a higher decay rate of microorganisms in bulk sludge. The group of Euryarchaeota played a major role in methane-producing and -enriched bulk sludge [57].

*SJA-15* and *Anaerolineaceae* affiliated with the phylum Chloroflexi were the most abundant families in the cake layer, which had a percentage of 15.37 ± 0.19%. Yao, et al. [28] presented that the *SJA-15* and *Anaerolineaceae* possessed filamentous morphological traits. The enrichment of these filamentous bacteria was consistent with the observation of SEM (Figure 7d). The relative abundance of the *Aeromonadaceae* (Proteobacteria) was 2.79 ± 0.26% in bulk sludge and increased to 7.93 ± 0.12% in the cake layer. According to the previous report [58], glucose greatly stimulated the fermentation of *Aeromonadaceae* to produce acid. It is deduced that the enrichment of *Aeromonadaceae* in the cake layer played a vital role in the degradation of organic matters. It is worth noting that the hydrophilicity of the sludge was increased by the acidogens and might lead to severe membrane fouling [59], indicating that *Aeromonadaceae* partially caused the membrane fouling.

## 4. Conclusions

In this study, a novel AnGDMBR system was designed and operated with lower pressure, flux, and energy for over 40 days using the optimal support material (800 mesh nylon net). The reactor showed a higher COD removal rate (85.45 ± 7.06%) and retention rate (46.34 ± 16.50%) compared with previous studies. The addition of a suspended carrier was an effective method to control the DM formation and alleviate membrane fouling by mechanical scouring, and the total resistance (3.2 × 10^7^ m^−1^) was relatively lower at mesophilic condition, probably due to the less compact nature of the DM layers. The irreversible fouling, including the pore-blocking and organic-bound fouling, became severe in the subsequent operation, accounting for 24.8% of the total fouling resistance. The filamentous bacteria (i.e., *SGA-15* and *Anaerolineaceae*) and the acidogens (i.e, *Aeromonadaceae*) were found to greatly accumulate in the DM layers. In summary, the novel AnGDMBR with the addition of suspended carriers has a superior performance (higher organic removal and lower fouling rates), which can be beneficial for optimizing the configuration and operation of AnDMBRs. Reducing the accumulation and growth of irreversible foulants in AnDMBRs needs to be further addressed in the future.

## Figures and Tables

**Table 1:** Fouling resistance distribution of the DM layer in the AnGDMBR.

	R_m_ (×10^7^ m^−1^)	R_un_ (×10^7^ m^−1^)	R_ir_ (×10^7^ m^−1^)	R_c_ (×10^7^ m^−1^)	R_t_ (×10^7^ m^−1^)
AnGDMBR ^a^	0.2 (6.2%)	0.08 (2.5%)	0.6 (24.8%)	2.14 (66.5%)	3.22 (100%)

^a^ The percentage of fouling resistance was shown in parentheses. R_un_ denotes the unrecoverable fouling after chemical cleaning and R_ir_ denotes the irreversible fouling including the pore blocking and organic-bounding fouling.

## Data Availability

The data presented in this study are available on request from the corresponding author.

## Supplementary Materials

The following are available online at https://www.mdpi.com/article/10.3390/membranes12070683/s1, Table S1. Comparison of the configuration, operation, and performance of the current work and other recent studies; Table S2. Details of the support materials; Table S3. Components of the synthetic wastewater; Table S4. Distribution of fouling resistances of different supporting materials; Table S5. Effluent quality of 50 g non-woven fabric and 800 mesh nylon net with five fouling-physical cleaning cycles; Table S6. Residual foulants amount of 50 g non-woven fabric and 800 mesh nylon net with five fouling-physical cleaning cycles; Table S7. Performance of the AnGDMBR; Table S8. Influent, supernatant, cake layer, and effluent fluorescence parameters. Figure S1. Experimental setup of dead-end filtration; Figure S2. The pictures of the AnGDMBR setup and membrane module; Figure S3. Particle size distribution of the bulk sludge in the AnGDMBR; Figure S4. Surface conditions of different support materials after cleaning at different stages; Figure S5. Total fouling resistances for 50 g non-woven fabric and 800 mesh nylon net.
