# Supplementary material for: A Novel Anaerobic Gravity-Driven Dynamic Membrane Bioreactor (AnGDMBR): Performance and Fouling Characterization
Source: Membranes (Basel). 2022 Jun 30;12(7):683. doi: 10.3390/membranes12070683 (PMC9351681; doi:10.3390/membranes12070683)
Supplement: Supplementary file 1 [file membranes-12-00683-s001.zip › membranes-1769525-supplementary.pdf]

Table S1. Comparison of the configuration, operation and performance of the current work and other recent studies.

Reactor configuration	Module configuration	Filtration mode	Support material	Pore size (μm)	Temperature (°C)	Membrane total resistance (m^{-1})	COD removal (%)	COD rejection (%)	Membrane fouling rate	The range of TMP and flux	Membrane cleaning methods	Reference
CSTR/ submerged	Flat-sheet	Gravity-driven	Nylon net	50	37	1.3×10^{11}	–	56.5-64.5	0.04 ($L/(m^2\cdot h^2)$)	– 2-11.5 LMH	Air back flushing	[1]
CSTR/ submerged	Tubular	Gravity-driven	Nylon net	P1: 200 P2: 52	26.3-26.7	–	–	–	P1: 659 (Pa/h) P2: 830 (Pa/h)	0-30 kPa 5-30 LMH	Surface brushing	[2]
UASB/ submerged	Tubular	Pump-driven	Nylon polyester net	P1: 70 P2: 10	35	P1: – P2: $9.2–37.9\times10^{10}$	P1: 78-96 P2: 69-90	–	P1: – P2: 0.6 ($L/(m^2\cdot h^2)$)	0-33 kPa 12.79-55.4LMH	Tap water	[3]
UASB/ submerged	Flat-sheet	Pump-driven	Nylon net	75	20-25	–	70.6–77.3	13.0-21.1	15.8-87.5 (Pa/h)	0-37 kPa 22.5-180LMH	Hydraulic cleaning and surface brushing	[4]
CSTR/ submerged	Flat-sheet	Pump-driven	Nylon net	20	31-35	–	91	15	413 (Pa/h)	0-50 kPa 4-13LMH	Biogas backwash	[5]
CSTR/ submerged	Flat-sheet	Gravity-driven	Nylon net	75	–	$0.65-1.75\times10^{11}$	88.0-90.2	–	24.2-24.4 ($L/(m^2\cdot h^2)$)	<10 kPa 140-1620LMH	Air back flushing and surface brushing	[6]
CSTR/ submerged	Tubular	Pump-driven	Stainless steel net	48	R1: 20-25 R2: 37	R1: 7×10^{11} R2: 8×10^{11}	97.0-99.2	–	R1: 43.4 (Pa/h) R2: 28.6 (Pa/h)	2-5 kPa 2.5-4.3 LMH	Air back flushing	[7]
CSTR/ inserted	Flat-sheet	Gravity-driven	Nylon net	18	33	3.2×10^{7}	78.4-92.5	29.8-62.9	23.5 (Pa/h)	0-0.8 kPa 3-7 LMH	Mechanical stirring with carrier	This study

Table S2. Details of the support materials.

Support material	Support material yarn type	Average pore size (μm)
30 g non-woven fabric	staple	35
50 g non-woven fabric	staple	30
70 g non-woven fabric	staple	25
600 mesh nylon net	mono-monofilament filter cloth	23
800 mesh nylon net	mono-monofilament filter cloth	18
1000 mesh nylon net	mono-monofilament filter cloth	13

Table S3. Components of the synthetic wastewater (all the reagents and chemicals were analytical reagent from the Sinopharm Group, China).

Potion	The concentration of the pollutant (12 L)	Formula	The concentration of the pollutant
$NaHCO_3$	15 g		
Concentrated solution	55 mL	Peptone	20.5 (g/L)
		Beef paste	7.0 (g/L)
		Glucose	70.0 (g/L)
		KH_2PO_4	5.0 (g/L)
		NH_4Cl	38.4 (g/L)
Trace element	10 mL	$FeSO_4{\cdot}7H_2O$	0.50 (mg/L)
		$MnCl_2{\cdot}4H_2O$	0.12 (mg/L)
		$ZnSO_4{\cdot}7H_2O$	0.25 (mg/L)
		$NaMnO_4{\cdot}6H_2O$	0.38 (mg/L)
		$CoCl_4{\cdot}6H_2O$	0.26 (mg/L)
		$NiCl_2{\cdot}6H_2O$	0.08 (mg/L)
		$CuSO_4{\cdot}5H_2O$	0.18 (mg/L)
		$CaCl_2$	0.88 (mg/L)
		H_3BO_3	0.12 (mg/L)
		$MgCl_2{\cdot}6H_2O$	0.26 (mg/L)

Table S4. Distribution of fouling resistances of different supporting materials.

	Rc (m^{-1})	Rb (m^{-1})	Rf (m^{-1})	Rm (m^{-1})	Rt (m^{-1})	Rf/Rt
**30g non-woven fabric**	3.69E+10	1.28E+04	2.12E+04	1.86E+06	3.69E+10	5.74E-07
**50g non-woven fabric**	2.22E+13	2.60E+04	3.32E+04	1.88E+06	2.22E+13	1.50E-09
**70g non-woven fabric**	3.21E+13	1.46E+05	3.62E+05	1.91E+06	3.21E+13	1.13E-08
**600 mesh nylon net**	1.16E+09	1.22E+04	2.06E+04	1.60E+06	1.16E+09	1.77E-05
**800 mesh nylon net**	2.06E+13	2.50E+04	2.80E+04	1.94E+06	2.06E+13	1.36E-09
**1000 mesh nylon net**	2.62E+14	3.29E+04	6.47E+04	2.01E+06	2.62E+14	2.47E-10

Table S5. Effluent quality of 50g non-woven fabric and 800 mesh nylon net with five fouling-physical cleaning cycles.

Items	Cycles	Polysaccharide (mg/L)	Protein (mg/L)	COD (mg/L)	Rejection rate (%)		
					Polysaccharide	Protein	COD
**Supernatant of sludge**		23.81	12.89	242.52			
**50g non-woven fabric**	1	14.70	6.16	87.03	38.25	52.21	64.11
	2	12.34	6.15	81.27	48.15	53.39	66.49
	3	11.98	5.73	78.39	49.66	55.55	67.68
	4	11.74	5.72	75.51	50.67	55.62	68.86
	5	9.73	5.23	61.12	59.11	59.43	74.80
**800 mesh nylon net**	1	11.98	3.96	78.39	49.66	69.28	67.68
	2	11.27	3.79	66.88	52.64	70.60	72.42
	3	9.73	3.62	64.00	59.11	71.92	73.61
	4	9.50	3.54	58.24	60.08	72.54	75.99
	5	8.91	3.37	49.60	62.55	73.86	79.55

Table S6. Residual foulants amount of 50g non-woven fabric and 800 mesh nylon net were operated with five fouling-physical cleaning cycles.

Items	Cycles	SS in permeate (g)	SS in cake layer (g)	The total SS (g)	Residual SS in the pore (g)
**Supernatant of sludge**		0.1239	0	0.1239	0
**50g non-woven fabric**	1	0.0579	0.0614	0.1193	0.0046
	2	0.0564	0.0622	0.1186	0.0053
	3	0.0561	0.0621	0.1182	0.0057
	4	0.0558	0.0622	0.1180	0.0059
	5	0.0552	0.0635	0.1187	0.0052
**800 mesh nylon net**	1	0.0570	0.0646	0.1216	0.0023
	2	0.0534	0.0683	0.1217	0.0022
	3	0.0519	0.0684	0.1203	0.0036
	4	0.0513	0.0689	0.1202	0.0037
	5	0.0504	0.0699	0.1203	0.0036

Table S7. Performance of the AnGDMBR.

Samples	Items	Concentration
B.S.	TSS(g/L)	3.78±0.10
C.L.	TSS(g/m^2)	0.66±0.03
Inf. (mg/L)	COD	464.73±17.31
	PS	330.79±5.25
	PN	452.77±1.83
Sup. (mg/L)	COD	136.67±68.33
	PS	24.48±6.20
	PN	19.71±7.76
Eff. (mg/L)	COD	67.74±33.24
	PS	6.10±4.36
	PN	7.19±5.95
EPS (mg/L)	PS	22.67±7.61
	PN	16.93±6.86
Removal rate (%)	COD	85.45±7.06
	PS	98.15±1.33
	PN	83.32±13.62
Retention rate (%)	COD	46.34±16.50
	PS	75.24±17.35
	PN	66.39±17.66

Table S8. Influent (Inf.), supernatant (Sup.), cake layer (C.K.) and effluent (Eff.) fluorescence parameters.

Peak location Ex (nm)/Em(nm)		Substance	Fluorescence Intensity			
			Inf.	Sup.	C.K.	Eff.
A	230/ 335-340	Aromatic protein	3539	1006	2107	1358
B	280/ 335-340	Tryptophan protein-like	6774	2074	2593	1367
C	330-335/ 400-420	Humic acidlike	616.3	688.4	317.4	714.4
D	238-240/ 415-435	Fulvic acidlike	340	442	344	670

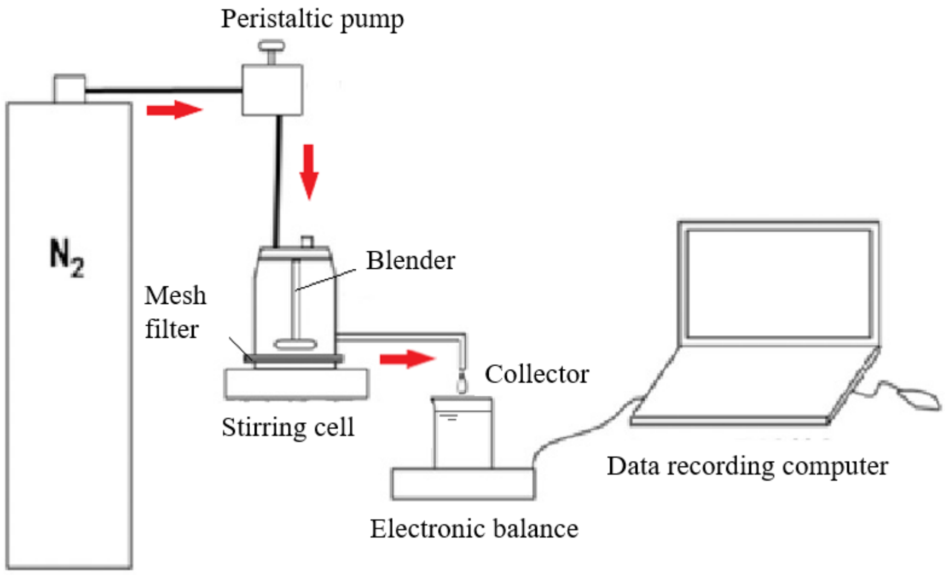

Figure S1. Experimental setup of dead-end filtration.

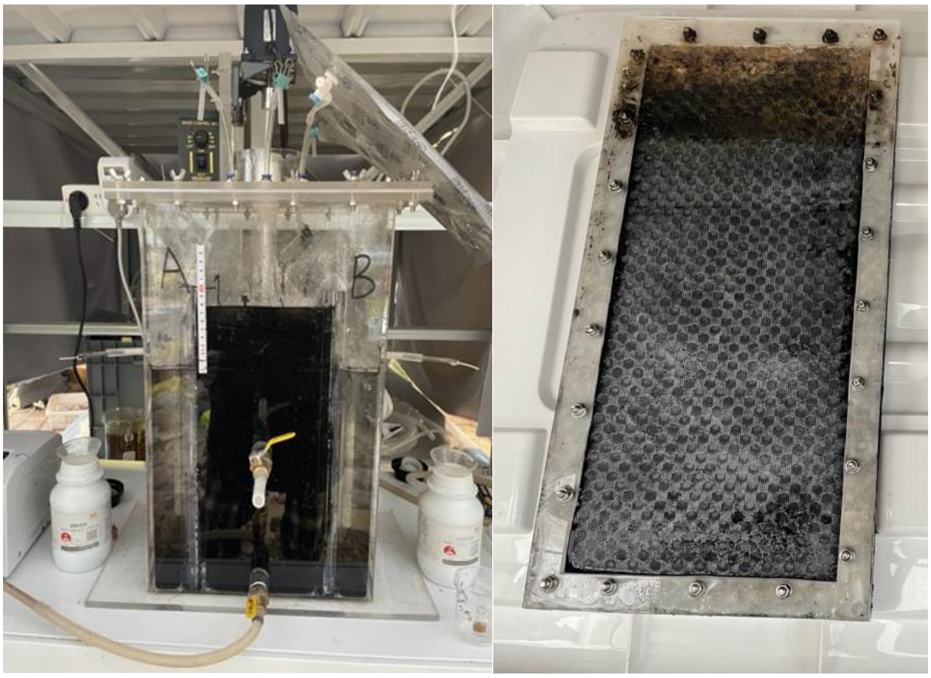

Figure S2. The picture of the AnGDMBR setup and membrane module.

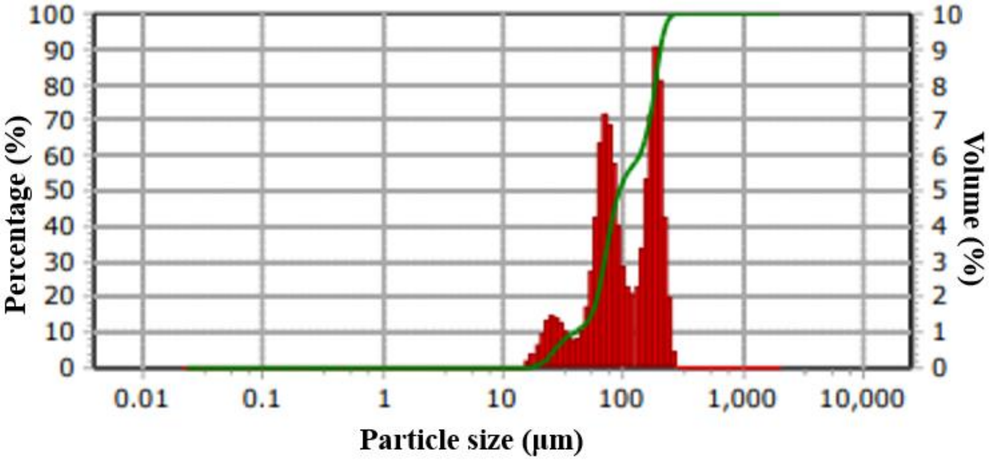

Figure S3. Particle size distribution of the bulk sludge in the AnGDMBR.

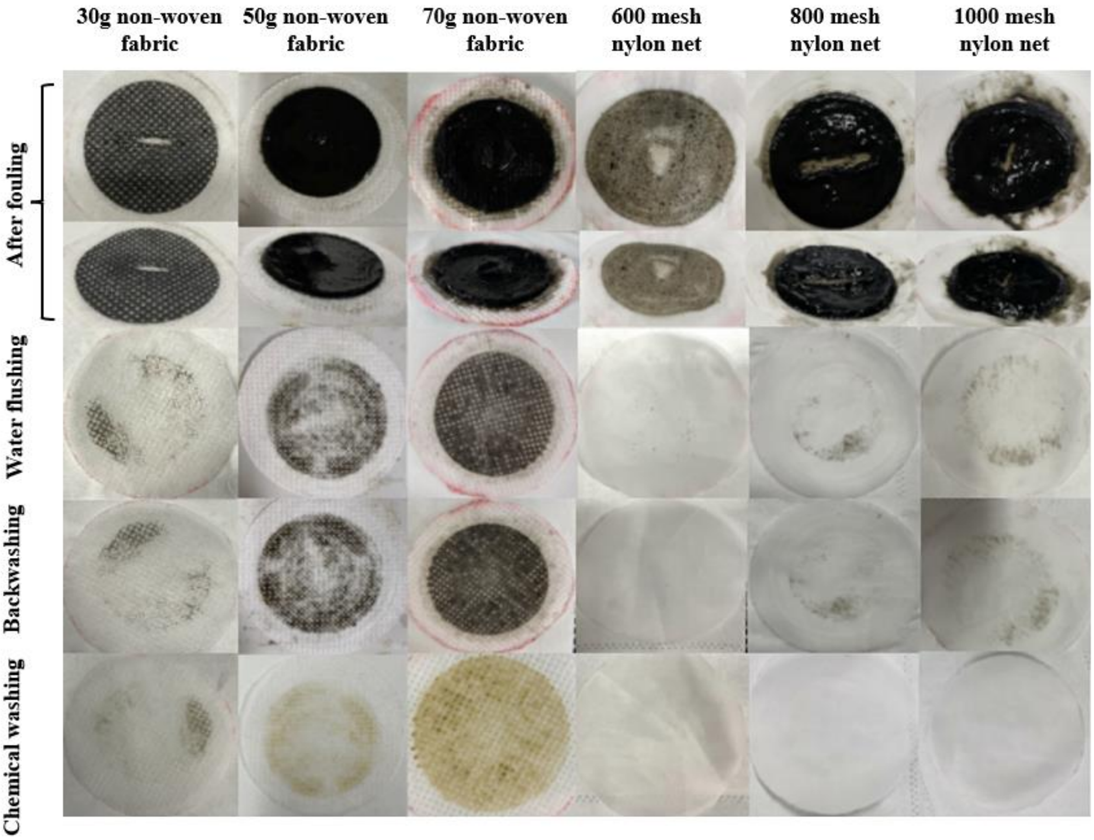

Figure S4. Surface conditions of different support materials after cleaning at different stages.

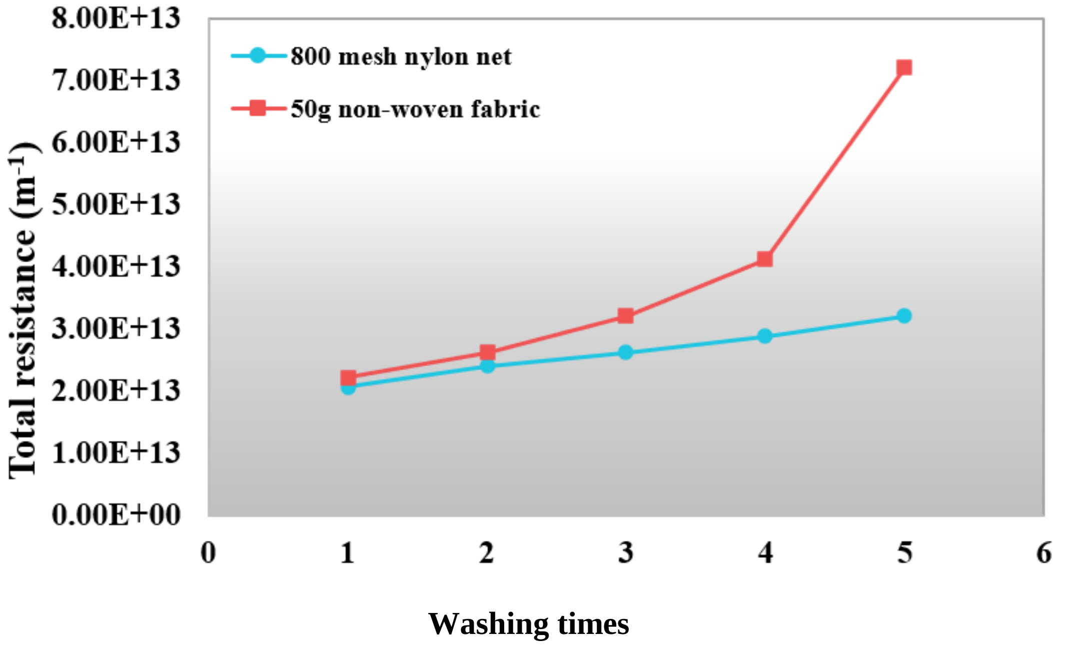

Figure S5. Total fouling resistances for 50g non-woven fabric and 800 mesh nylon net.

References

1. Tang, J.; Wang, X.C.; Hu, Y.; Ngo, H.H.; Li, Y. Dynamic membrane-assisted fermentation of food wastes for enhancing lactic acid production. *Bioresour. Technol.* **2017**, *234*, 40-47. doi:https://doi.org/10.1016/j.biortech.2017.03.019.
2. Saleem, M.; Masut, E.; Spagni, A.; Lavagnolo, M.C. Exploring dynamic membrane as an alternative for conventional membrane for the treatment of old landfill leachate. *J Environ Manage.* **2019**, *246*, 658-667. doi:https://doi.org/10.1016/j.jenvman.2019.06.025.
3. Paçal, M.; Semerci, N.; Çallı, B. Treatment of synthetic wastewater and cheese whey by the anaerobic dynamic membrane bioreactor. *Environ. Sci. Pollut. Res.* **2019**, *26*, 32942-32956. doi:10.1007/s11356-019-06397-z.
4. Yang, Y.; Zang, Y.; Hu, Y.; Wang, X.C.; Ngo, H.H. Upflow anaerobic dynamic membrane bioreactor (AnDMBR) for wastewater treatment at room temperature and short HRTs: Process characteristics and practical applicability. *Chem. Eng. J.* **2020**, *383*, 123186. doi:https://doi.org/10.1016/j.cej.2019.123186.
5. Yurtsever, A.; Basaran, E.; Ucar, D.; Sahinkaya, E. Self-forming dynamic membrane bioreactor for textile industry wastewater treatment. *Sci Total Environ.* **2021**, *751*, 141572. doi:https://doi.org/10.1016/j.scitotenv.2020.141572.
6. Yang, Y.; Deng, W.; Hu, Y.; Chen, R.; Wang, X.C. Gravity-driven high flux filtration behavior and microbial community of an integrated granular activated carbon and dynamic membrane bioreactor for domestic wastewater treatment. *Sci Total Environ.* **2022**, *825*, 153930. doi:https://doi.org/10.1016/j.scitotenv.2022.153930.
7. Pan, W.; Ouyang, H.; Tan, X.; Deng, R.; Gu, L.; He, Q. Anaerobic dynamic membrane bioreactors for synthetic blackwater treatment under room temperature and mesophilic conditions. *Bioresour. Technol.* **2022**, *355*, 127295. doi:https://doi.org/10.1016/j.biortech.2022.127295.
